# Thermal Image Super-Resolution Based on Lightweight Dynamic Attention Network for Infrared Sensors

**DOI:** 10.3390/s23218717

**Published:** 2023-10-25

**Authors:** Haikun Zhang, Yueli Hu, Ming Yan

**Affiliations:** School of Mechatronic Engineering and Automation, Shanghai University, Shanghai 200444, China; haikunzhang@shu.edu.cn (H.Z.); shuforyanming@foxmail.com (M.Y.)

**Keywords:** infrared sensor, thermal image, super-resolution, attention mechanism, dynamic network

## Abstract

Infrared sensors capture infrared rays radiated by objects to form thermal images. They have a steady ability to penetrate smoke and fog, and are widely used in security monitoring, military, etc. However, civilian infrared detectors with lower resolution cannot compare with megapixel RGB camera sensors. In this paper, we propose a dynamic attention mechanism-based thermal image super-resolution network for infrared sensors. Specifically, the dynamic attention modules adaptively reweight the outputs of the attention and non-attention branches according to features at different depths of the network. The attention branch, which consists of channel- and pixel-wise attention blocks, is responsible for extracting the most informative features, while the non-attention branch is adopted as a supplement to extract the remaining ignored features. The dynamic weights block operates with 1D convolution instead of the full multi-layer perceptron on the global average pooled features, reducing parameters and enhancing information interaction between channels, and the same structure is adopted in the channel attention block. Qualitative and quantitative results on three testing datasets demonstrate that the proposed network can superior restore high-frequency details while improving the resolution of thermal images. And the lightweight structure of the proposed network with lower computing cost can be practically deployed on edge devices, effectively improving the imaging perception quality of infrared sensors.

## 1. Introduction

RGB sensors are universally used in smartphones, drones, laptops, and other devices due to their excellent imaging quality and speed. However, the image quality captured by RGB sensors degrades dramatically under harsh conditions [1]. The wavelength of long-wave infrared radiation ranges between 7 and 14 μm, while the wavelength of visible light in the electromagnetic spectrum lies between 390 and 780 nm. Therefore, infrared sensors have a robust ability to penetrate smoke, fog, and haze, and can replace RGB sensors in the aforementioned scenarios. Nonetheless, high-resolution (HR) infrared focal plane arrays are expensive, and civilian uncooled infrared detectors output low-resolution (LR) thermal images that cannot match the megapixel RGB sensors [2]. The straightforward approach is to design complex hardware devices to enhance the resolution of thermal images, but the extended manufacturing time limits their practical application. In recent years, a method known as super-resolution (SR) has been extensively developed to enhance LR image resolution [3].

The purpose of image super-resolution (ISR) is to recover the corresponding HR images from the observed degraded LR counterparts. The current ISR methods can be classified into three categories based on technical approaches: interpolation based, reconstruction based, and learning based. The linear interpolation algorithms (e.g., nearest, bilinear, and bicubic) design interpolation weighting functions based on the assumption of the local smoothness of the image [4]. They are simple and fast but are prone to aliasing artifacts in areas with rich high-frequency information. To overcome the shortcomings of linear interpolation, more works focus on nonlinear interpolation algorithms, i.e., adaptive image interpolation. For example, adaptive interpolation methods based on edge guidance [5,6] or perception [7] and non-local means [8] improve the perceptual quality of interpolated images, but high-frequency details are still insufficient under large scale factors. The ISR methods based on pixel domain reconstruction employ the introduced prior knowledge as a constraint to iteratively solve the objective function until it converges to the local optimal solution. Projection onto convex sets, iterative back projection and maximum a posteriori (MAP) are representative algorithms [4]. The reconstruction-based ISR methods [4,9,10] first establish a degradation model from HR images to LR images, which is the inverse problem of ISR [11,12,13]. Therefore, the degradation model can be reversely solved based on algorithms such as MAP estimation, and the HR image can be predicted. For instance, Greenbaum et al. [13] generated HR images based on super-resolved stacks of multiple shifted LR images to improve the field-of-view of dense samples captured by lensfree holographic microscopy. As a shallow learning algorithm based on learning, traditional sparse dictionary-based ISR methods suffer from slow speed and are limited by the size of the overcomplete dictionary, resulting in unsatisfying performance [14]. However, with the development of multimedia technology, the above methods have gradually reached performance bottlenecks and cannot meet the needs of generating high-definition or even ultra-high-definition images. Recently, ISR models based on deep convolutional neural networks (CNNs) achieved impressive performance gains over traditional methods [1,3]. CNN-based ISR methods map LR images to HR images in an end-to-end manner according to the datasets during training. Taking advantage of the powerful nonlinear fitting and automatic feature learning capabilities of CNNs, as well as the emergence of dedicated acceleration hardware such as the neural processing unit (NPU), the performance of the ISR network trained with massive training data is significantly better than the above traditional methods. SRCNN [15,16], as the first CNN-based ISR model, exhibits significantly superior performance than interpolation- and sparse coding-based methods. Relying on the powerful nonlinear fitting ability of CNN, CNN-based ISR models for RGB sensors have been continuously proposed [17]. However, there are relatively few ISR methods specifically designed for infrared sensors. Therefore, there is an urgent need to develop a thermal image SR model based on CNN that can be applied in practice.

Inspired by the human visual system (HVS), progressively ISR networks employ attention mechanisms to improve performance [18]. The HVS is not equally capable of processing all the information contained in the observed scene but concentrates limited computing resources on the most information-rich regions. For instance, when humans gaze at the sky, their attention is likely to be focused on objects like birds flying in the sky rather than the background of the sky itself. Similarly, it is the high-frequency details such as edges and textures rather than smooth areas that most affect the perceived quality of an image. The purpose of ISR is to maximize the recovery of high-frequency information while improving the resolution. Channel attention (CA) and spatial attention (SA) mechanisms are currently widely used attention methods and have been proven to improve many low-level computer vision tasks, including ISR [19,20,21]. CA and SA perform recalibration operations in the channel and pixel spaces of feature maps, respectively. To make full use of channel and spatial information interaction, many works stack CA and SA blocks to form attention modules and reuse them [1,22,23]. Previous studies [18,24] have shown that although LR images have insufficient resolution, they still contain a large amount of low-frequency information and high-frequency details. ISR networks that without attention blocks process all channels and image regions equally cannot effectively recover high-frequency details. Therefore, the attention mechanism can enrich the edges and texture details of the final super-solved HR image, thereby improving the perceptual quality.

The few existing CNN-based thermal image SR models for infrared sensors mainly suffer from the following three shortcomings. (1) **Inefficient sequential stacking of attention modules**. As shown by Chen et al. [18], simply using the same attention module hierarchy to extract features is not always beneficial to the final RGB ISR performance. And we prove in Section 3 that thermal image SR for infrared sensors should embrace attention modules variously at different depths of the network. The results show that the early attention feature extraction modules enhance low-level information, the tail modules extract high-frequency details, and the middle attention modules enhance the above two features mixedly. Therefore, it is necessary to dynamically adjust the attention weights according to the characteristics of different stages of the network. (2) **Networks are complex and unwieldy**. With the advent of residual structures [25], it is possible to stably train very deep networks. Since then, more and more ISR networks have improved performance by continuously increasing network capacity (i.e., increasing the number of layers of the network and the width of each layer). For example, EDSR [26] has more than 40M parameters, and the huge computing power requirement makes it almost impossible to deploy in embedded devices, which limits its practical application. Although well-designed networks with more parameters can continuously improve performance, the resulting gains and training/inference costs have to be considered. In other words, there should be a trade-off between the performance and scope of parameters. (3) **The designed attention module is not compact**. Inspired by SENet [27], more researchers [28,29,30] have invested in designing complex CA structures to enhance channel dimension features extraction, or combining convoluted SA blocks to improve performance. Although these methods enrich the details of the super-solved images, the heavy computational burden leads to slow inference, limiting their practical applications. Our initial idea was to design an efficient attention module that could reduce the complexity of the network and achieve satisfactory performance, making it possible to deploy on edge devices.

To address the above issues, we propose a lightweight dynamic attention super-resolution network (LDASRNet) for infrared sensors to super-resolve LR thermal images. The LDASRNet consists of a shallow feature extraction (SFE) module, a deep feature extraction (DFE) module and a feature reconstruction (FRec) module. The SFE module consists of only one convolutional layer with filter size 3 × 3, which is used to extract shallow features. The DFE module consists of sequentially stacked dynamic attention blocks (DABs). Different from previous works, our proposed DAB adaptively and dynamically assigns weights to attention and non-attention branches according to different deep features of the network. In particular, the efficient CA block efficiently aggregates channel dimension features. And the pixel attention block generates 3D instead of 2D attention maps compared with SA, and obtains more performance gains with less computational cost. The FRec module is used to reconstruct the final HR thermal image.

In this paper, our main contributions are as follows:

- Aiming at the problem of the low perception quality of civilian LR uncooled infrared sensors, we propose a CNN-based dynamic attention network to super-resolve LR thermal images. The proposed network outperforms compared models with more parameters while maintaining a lightweight structure, showing the potential to be deployed on a handheld thermal imaging camera devices with limited computing power. We train the proposed network with a mixture of data augmentation methods, and experiments show that multiple pixel domain data augmentation methods can effectively improve the ISR performance.
- We propose a lightweight DAB. DAB at different stages dynamically reweights the attention and non-attention branches according to the input feature maps. Furthermore, our proposed attention branch consists of an efficient CA block and a residual pixel attention block; the latter differs from existing SA mechanisms that only generate 2D attention maps but use 3D attention maps to enhance spatial features.
- Qualitative and quantitative results on multiple datasets demonstrate that the proposed network can effectively improve thermal image resolution while recovering visually pleasing high-frequency details. Specifically, the proposed network achieves the highest performance metrics on the public testing dataset with ×3 scale factor. And compared to the second-best model $A^{2}N$, the scope of parameters is only 34% of latter (i.e., 0.34 M vs. 1.0 M).

The remainder of this paper is organized as follows. Section 2 describes the technical details of the existing attention mechanism, as well as CNN-based SR methods for RGB and thermal images. Section 3 shows the motivation and necessity of our proposed dynamic attention for thermal image SR. Section 4 details our proposed dynamic attention network structure. Section 5 qualitatively and quantitatively compares the proposed network with state-of-the-art lightweight ISR models. We conclude this paper in Section 6.

## 2. Related Work

### 2.1. CNN-Based Image Super-Resolution

Traditional ISR methods based on the sparse coding framework represent HR image patches as sparse linear combinations of atoms in an over-complete dictionary [14]. Therefore, the ISR performance is limited by the dictionary size, and the inference speed is slow, resulting in dissatisfactory ISR performance under large scale factors [1].

As CNNs have demonstrated impressive accuracy in the field of image recognition [31], CNN-based ISR methods have emerged. SRCNN [15], as a pioneering CNN-based ISR work, achieves significantly superior performance than traditional ISR methods. And FSRCNN [16] further boosts SRCNN to obtain more gains at a lower computational cost. However, SRCNN only has three convolutional layers, resulting in a relatively small model capacity, which may not be able to recover sufficient details when faced with large-scale upsampling scale factors. Following SRCNN, VDSR [32] explores multi-layer small-size convolution kernels to expand the receptive field, and the resulting 20-layer network greatly improves accuracy. As the residual connection in ResNet [25] makes it possible to stably train very deep networks, the residual structure is widely used in the ISR models. Unlike SRResNet [33], EDSR [26] consists of 32 residual blocks that remove batch normalization, reducing memory consumption and artifacts. However, the above model adopts the pre-upsampling method, that is, the LR input is first interpolated to the desired size, which increases the difficulty and computational burden of subsequent deep feature extraction. Therefore, more works adopt a post-upsampling structure, which upsample features to the expected resolution at the end of the model [3]. RDN [34] employs residual blocks with dense connections to make full use of the features of previous layers, and the formed persistent memory mechanism effectively utilizes abundant features. Inspired by SENet [27], RCAN [24] models interactions between channels to recalibrate the channel features. As a scale-attention-based network, RCAN adopts a two-level residual CA structure to deepen the network depth while utilizing more low-frequency information. Similarly, HAN [35] with channel–spatial attention modules to explore the relationship between pixel domain and channel dimension, and uses layer attention to utilize the output features of all residual groups. The effectiveness of CA and SA to reweight features in channel and spatial dimensions respectively to enhance high-frequency information has been proven, and extensively adopted in low-level (e.g., ISR) and high-level computer vision tasks [35,36,37,38].

Motivated by ISR in the visible spectral domain, Choi et al. [39] proposed TEN, a network consisting of four convolutional layers for end-to-end mapping of LR thermal images. Limited by the expensive HR thermal detectors at that time, it was difficult to obtain a large number of paired LR-HR thermal images, TEN used RGB images as the training dataset. Similarly, Marivani et al. [40] proposed multimodal SR models DMSC and ${\mathrm{DMSC}}_{+}$, using HR RGB images as an auxiliary to super-resolve LR near-infrared thermal images. However, Rivadeneira et al. [41] demonstrated that a network trained with thermal images is superior for thermal image SR inference compared to RGB training datasets. In addition, the authors also constructed a dataset consisting of 101,640 × 512 resolution thermal images to activate the research in the field of thermal image enhancement. Aiming at the problem of severe thermal image noise caused by high clutter in the maritime environment, Bhattacharya et al. [42] proposed two CNN-based networks to perform denoising and SR tasks, respectively, to improve the perception of maritime thermal images. The idea of cascading two networks is also adopted in CDN_MRF [43]. The first residual network of CDN_MRF is used to extract thermal image structure, and the second network is used for fine high-frequency details. As the champion of the Perception Beyond the Visible Spectrum (PBVS)-2020 Thermal Image SR Challenge, the progressive feature extraction module of the TherISuRNet [2] was adopted to generate HR thermal images. In order to reduce redundant features extraction in the deep networks, ChaSNet [44] with the channel separation method eliminates overload features in the trunk of the network. However, the limited receptive fields of the convolution kernels adopted in the above models limit the performance. Zhang et al. [1] proposed MPRANet, a thermal image network composed of residual blocks with parallel convolution kernels of different sizes to effectively extract local and global features.

Apart from discriminative models, some research efforts have focused on generative models, such as generative adversarial networks (GANs) [45] for thermal image SR. Liu et al. [46] integrated the gradient prior knowledge of natural scenes, and trained the GAN-based thermal image SR network with RGB images as style feature auxiliary information. Rivadeneira et al. [47] proposed CycleGAN, a network based on [48] and with an unsupervised training method. In general, GAN-based thermal image SR model training is unstable and prone to mode collapse, so most thermal image SR methods are still based on CNN.

### 2.2. Attention Mechanisms in Image Super-Resolution

ISR networks extensively employ CA and SA modules to enhance channel and spatial dimension feature maps, respectively. These two attention paradigms focus on rich patterns to reconstruct HR images.

**Figure 1:**
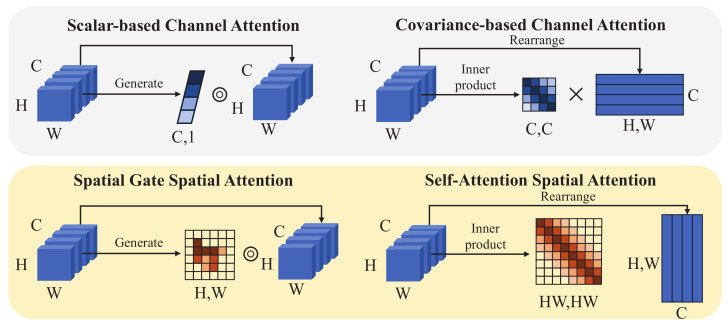
Schematic diagram of channel attention and spatial attention in different paradigms.

**Channel Attention**. Channel attention is divided into scalar-based CA [27] and covariance-based CA [49]. The schematic diagram of the two paradigms is shown in Figure 1. Scalar-based CA generates weights in the channel dimension for reweighting feature maps. This means that all pixels of each channel are rescaled by the same scalar, i.e., CA is the channel dimension isotropy operator. Whereas covariance-based CA performs inner product (i.e., self-attention) operation on the input features to generate a cross-covariance matrix to transfer information between channels.

**Spatial Attention**. Spatial attention can be viewed as an anisotropic operator, i.e., each pixel of all channels is multiplied by various weights to highlight features. Spatial gate-based [29,30] and self-attention-based [50,51] SA are two representative paradigms. As shown in Figure 1, the spatial gate SA generates channel-independent weight masks, while the self-attention SA computes the cross-covariance in the pixel domain. The information interaction of the above two SAs is only in the spatial dimension, and there is no interaction between the channels.

As shown in Figure 1, the CA operation is isotropic in the spatial dimension, that is, the computational complexity of CA is less than that of SA. Therefore, lightweight ISR networks tend to adopt CA rather than SA. However, some research works [1,21] have shown that the combined of CA and SA can help improve ISR performance. The aggregation capabilities of CA and SA in different feature dimensions complement each other. We propose to use pixel-wise attention, which is more effective than SA, and combined with CA, to improve performance while maintaining a compact network structure.

## 3. Motivation

According to ISR research works on RGB sensors [18], LR images are mixed with low-frequency information and high-frequency details, such as edges and textures. A network without incorporating an attention mechanism handles all frequency bands equally at all layers, resulting in inefficient redundant computation. The attention mechanism can enhance high-frequency features and improve visual quality. However, there are few studies on thermal image SR task for infrared sensors to prove the above assumptions.

**Figure 2:**
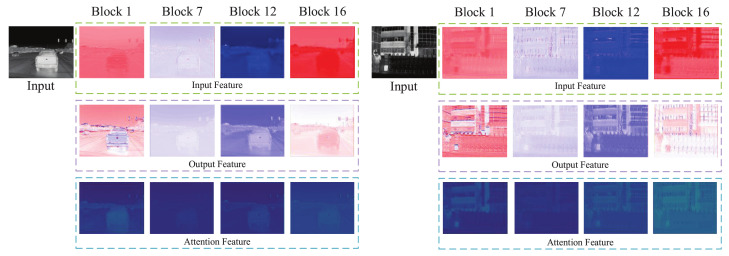
Visualization of feature maps. In the input and output feature maps, white, red, and blue pixels indicate zero, positive, and negative values, respectively. The brighter the pixels in the attention maps, the larger the value of the attention coefficients.

We construct a network composed of attention modules to explore the properties of the attention mechanism in thermal image SR task. The LR thermal image first passes through a convolutional layer to extract shallow features, then sequentially extracts deep features by 16 attention blocks, and finally outputs the HR thermal image through an upsampling module. The proposed attention module consists of sequential channel and spatial attention blocks such that each pixel in the feature map can be rescaled independently. We visualize the feature maps of the outputs of some attention modules as shown in Figure 2. As shown in Figure 2, the behaviors of attention modules at different depths are quite different, even diametrically opposite. The shallow attention modules extract low-frequency features, i.e., flat regions are enhanced, the tail attention modules enhance high-frequency information, and the middle modules mix the above two operations.

Furthermore, to verify whether all attention modules contribute to the final performance gain, we replace the attention modules of some layers with residual blocks, and the results are shown in Table 1. The peak signal-to-noise ratio (PSNR) and structural similarity index (SSIM) are performance metrics. Table 1 shows that the attention module actually improves the ISR accuracy, and the location of the attention module is critical to the performance. The pure residual block structure achieved the same performance as the structure with attention modules in the first half, and the quantitative comparison results between the network with attention blocks in the second half and the fully attention network were consistent. According to the above experiments, we can conclude the following conclusions: (1) It is beneficial to adopt the attention mechanism for ISR task. (2) It is not always optimal to embrace the attention blocks equally. Therefore, we propose a dynamic attention network for infrared sensors to super-resolve LR thermal images.

## 4. Proposed Method

**Figure 3:**
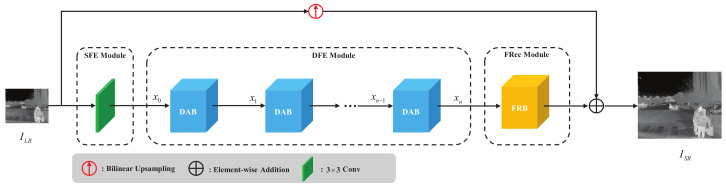
The structure of the proposed lightweight dynamic attention super-resolution network (LDASRNet).

We describe the details of the proposed lightweight dynamic attention super-resolution network (LDASRNet) for infrared sensors in this section. The structure diagram of LDASRNet is shown in Figure 3.

The LDASRNet consists of the following three modules:

(1) *Shallow feature extraction* (SFE) module. The input LR thermal image first passes through an SFE module consisting of a 3 × 3 convolution kernel to extract low-level features. Our experiments show that using a single 3 × 3 convolution is a acceptable balance between performance and parameters.
(2) *Deep feature extraction* (DFE) module. The output of the SFE module is used to extract deeper features through the DFE module. As a key component of LDASRNet, the DFE module consists of *K* dynamic attention blocks for dynamically enhancing high-frequency feature extraction according to the input feature maps.
(3) *Feature reconstruction* (FRec) module. The FRec module constructs the outputs of the DFE module into the final HR thermal image. Due to the lower LR spatial resolution of the ×4 scale factor compared to the ×2 and ×3 scale factors, reconstruction is more difficult. We designed two types of FRec modules specifically for the ×2/×3 and ×4 scale factors, respectively.

### 4.1. Shallow Feature Extraction Module

Given a LR thermal image $I_{LR}\in R^{C\times H\times W}$, where *C* is the number of channels, *H* and *W* are the height and width, respectively. The function of the SFE module can be expressed mathematically as:

(1) $$x_{0}=f_{SFE}\left(I_{LR}\right)$$

where $x_{0}$ is the output of the SFE module and $f_{SFE}\left(•\right)$ represents the SFE function, which consists of a single 3 × 3 convolution kernel. An alternative scheme is to utilize more convolutional layers to form the SFE module, but we found that this is inefficient and unnecessary. A single 3 × 3 convolutional layer can already extract low-frequency information well, and it balances ISR accuracy and computational burden.

### 4.2. Deep Feature Extraction Module

**Figure 4:**
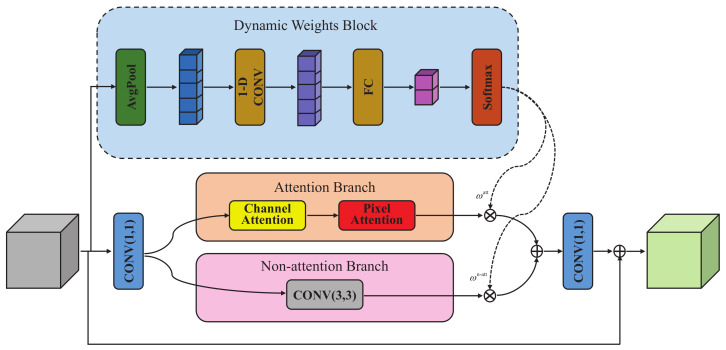
The overall structure of the dynamic attention block. **AvgPool** and **FC** represent global average pooling and full connection operations, respectively.

As shown in Figure 3, the outputs $x_{0}$ of the SFE module are then used to extract high-level patterns through a DFE module composed of *K* DABs. Each DAB consists of a dynamic weight block (DWB) as well as an attention branch and a non-attention branch. The structure of DAB is shown in Figure 4.

#### 4.2.1. Dynamic Weights Block

As mentioned in Section 3, simply stacking attention blocks failed lead to optimal performance gains. Therefore, we propose a dynamic attention mechanism to maximize the enhancement of high-frequency information. The specific implementation of the dynamic attention mechanism is shown in Figure 4.

The input $x_{i-1}\in R^{C_{i-1}\times H_{i-1}\times W_{i-1}}$ passes through the global average pooling (GAP) layer of *i*-th DAB to obtain a feature vector $z_{i-1}\in R^{C_{i-1}\times 1\times 1}$, where the *k*-th statistic of $z_{i-1}$ is calculated according to:

(2) $$\begin{array}{cc} z_{i-1}^{\left(k\right)} & =f_{avg}\left(x_{i-1}^{\left(k\right)}\right)\\ & =\frac{1}{H_{i-1}\times W_{i-1}}\sum_{m=1}^{H_{i-1}}\sum_{n=1}^{W_{i-1}}x_{i-1}^{\left(k\right)}\left(m,n\right) \end{array}$$

where $f_{avg}\left(•\right)$ is channel-wise GAP, $x_{i-1}^{\left(k\right)}\in R^{1\times H_{i-1}\times W_{i-1}}$ and $z_{i-1}^{\left(k\right)}\in R^{1\times 1\times 1}$ are the feature map of the *k*-th channel and its corresponding GAP output, respectively. There are many other sophisticated methods for aggregating global information; we use the simplest GAP to achieve this goal efficiently. Intuitively, the usual approach is to follow $z_{i-1}$ with two fully connected layers, namely a channel reduction layer and a channel increase layer, to enhance the information interaction between channels [27]. However, we use 1D convolution to simplify the above operation and achieve superior performance while reducing the number of parameters. We will demonstrate the necessity of choosing 1D convolution instead of two fully connected layers.

If we choose two fully connected layers, the output *w* can be expressed as:

(3) $$w=\sigma (f_{\left\{w_{1},w_{2}\right\}}\left(g\left(z_{i-1}\right)\right))$$

where $w\in R^{C_{i-1}\times 1\times 1}$, σ is a sigmoid function, and the specific form of $f_{\left\{w_{1},w_{2}\right\}}$ is:

(4) $$f_{\left\{w_{1},w_{2}\right\}}\left(y\right)=W_{2}\mathrm{RELU}\left(W_{1}y\right)$$

where RELU is the rectified linear unit [52]. As the weights of the channel reduction layer $W_{1}$ and channel increase $W_{2}$ have sizes $C\times \frac{C}{r}$ and $\frac{C}{r}\times C$ respectively, *r* is an adjustable attenuation parameter. The above operation reduces the parameter burden but destroys the direct one-to-one correspondence between channels and weights [53]. One weight element of a fully connected layer utilizes all channel information; however, the operation shown in Equation (4) first maps full-size features to a low-dimensional space, and then maps back to a high-dimensional space. The direct relationship between channels and weights is broken. We show that 1D convolution elegantly preserves the explicit correspondence between channels and weights.

We enhance the cross-channel interaction using 1D convolution as shown in the following equation:

(5) $$w=\sigma_{\mathrm{softmax}}\left(f_{\mathrm{FC}}\left(f_{{C1D}_{t}}\left(z_{i-1}\right)\right)\right)$$

where $f_{{C1D}_{t}}\left(•\right)$ represents a 1D convolution with filter size *t*, and $f_{\mathrm{FC}}\left(•\right)$ and $\sigma_{\mathrm{softmax}}\left(•\right)$ are fully connected layer and softmax functions, respectively.

Our goal is to generate two weights for the attention and non-attention branches respectively from the input feature maps. We use GAP based on the following two considerations: (1) As the depth of the network increases, GAP effectively increases the receptive field, thereby extracting global image information. (2) Compared with directly applying the fully connected layer, the GAP drastically reduces parameters, suppresses overfitting and can flexibly adapt to changes in the size of input feature maps. Therefore, the outputs of our proposed DWB block are:

(6) $$w^{\mathrm{attn}},w^{n-\mathrm{attn}}=\sigma_{\mathrm{softmax}}\left(f_{\mathrm{FC}}\left(\mathrm{RELU}\left(f_{{C1D}_{t}}\left(z_{i-1}\right)\right)\right)\right)$$

We use $w^{\mathrm{attn}}$ and $w^{n-\mathrm{attn}}$ to recalibrate the attention branch and no-attention branch, respectively. The above operation can be formulated as:

(7) $$x_{i}=f_{1\times 1}\left(w_{i-1}^{\mathrm{attn}}\times x_{i-1}^{\mathrm{attn}}+w_{i-1}^{n-\mathrm{ttn}}\times x_{i-1}^{n-\mathrm{attn}}\right)+x_{i-1}$$

where $w_{i-1}^{\mathrm{attn}}$ and $w_{i-1}^{n-\mathrm{ttn}}$ represent the weights of the attention branch and the non-attention branch, respectively. $x_{i-1}^{\mathrm{attn}}$ is the output of the attention branch, and $x_{i-1}^{n-\mathrm{attn}}$ is the output of the non-attention branch. $f_{1\times 1}\left(•\right)$ is a convolution layer with a convolution kernel size of 1 × 1.

In order to reduce the learning difficulty and compress the filter space, we let $w^{\mathrm{attn}}+w^{n-\mathrm{attn}}=1$, and the softmax function obtains the normalized weights.

The size of the 1D convolution kernel *t* determines the interaction range between local channels. In order to avoid manually determining *t*, which consumes time and resources, we automatically determine *t* in the following way:

(8) $$t=\phi \left(C_{i-1}\right)={\left|\frac{\log_{2}\left(C_{i-1}\right)}{\gamma }+\frac{b}{\gamma }\right|}_{odd}$$

where ${\left|z\right|}_{odd}$ represents the odd number closest to *z*, $C_{i-1}$ denotes the number of channels, γ and *b* are two hyper-parameters, and we empirically set γ=2 and b=1, respectively. We use nonlinear mapping instead of linear mapping to extend the representation capacity, and the local interaction range is proportional to the channel dimension size.

#### 4.2.2. Attention Branch

**Figure 5:**
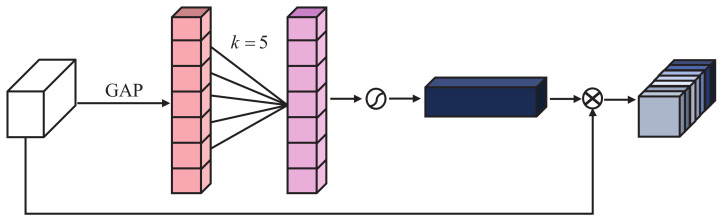
The overall structure of the channel attention block. **GAP** represents global average pooling, and k=5 represents the 1D convolution kernel size.

**Figure 6:**
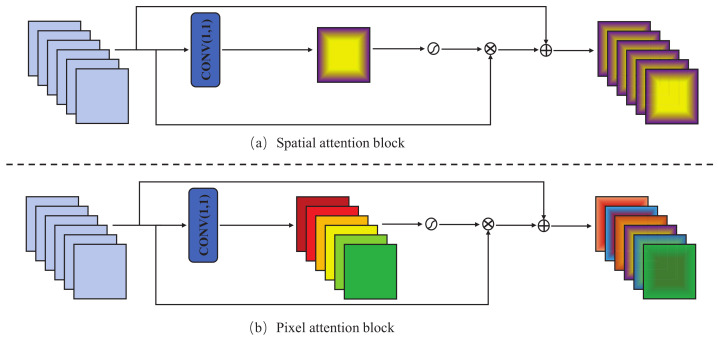
The overall structures of the spatial and pixel attention blocks. (**a**) Spatial attention block. (**b**) The proposed residual pixel attention block.

As shown in Figure 4, the attention branch consists of a channel attention block (CAB) and pixel attention block (PAB), and their structures are shown in Figure 5 and Figure 6b, respectively.

Similar to DWB, we use GAP to obtain channel-by-channel global information, and then perform cross-channel interaction with 1D convolution without channel dimensionality reduction. Our efficient CAB reduces model complexity while capturing local dependencies between channels.

The characteristics of feature map of each channel vary greatly. As shown in Figure 6a, the conventional spatial attention block equally weights all pixels of each channel feature map, which cannot fully enhance the spatial information. Inspired by PAN [54], we propose pixel attention with residual connections, which weights the pixels of each channel independently as shown in Figure 6b.

#### 4.2.3. Non-Attention Branch

As a complement, we introduce the non-attention branch to extract information ignored by the attention branch. We use a single 3 × 3 convolutional layer to form the non-attention branch. It is worth noting that non-attention branches with more complex structures can be adopted as alternatives, but 3 × 3 convolutional layers are suitable for our proposed lightweight structure.

To sum up, the output of the DFE module is:

(9) $$x_{n}=f_{DAB}^{K-1}\left(f_{DAB}^{K-2}\left(\cdots f_{DAB}^{1}\left(f_{DAB}^{0}\left(x_{0}\right)\right)\cdots \right)\right)$$

where $f_{DAB}^{i}\left(•\right)$, i=0,1,⋯,K−1, is the *i*-th DAB.

### 4.3. Feature Reconstruction Module

**Figure 7:**
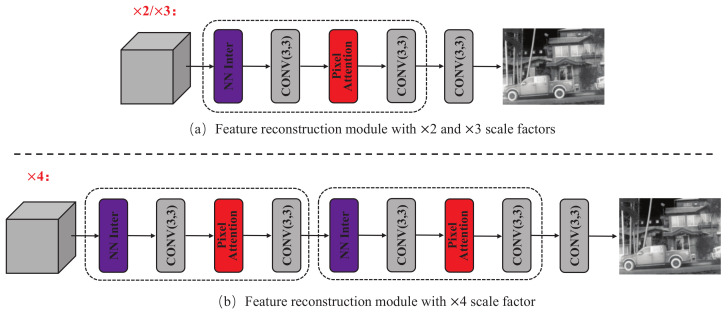
The overall structures of the feature reconstruction blocks. **NN Inter** is nearest neighbor interpolation operation. (**a**) Feature reconstruction block for ×2 and ×3 scale factors. (**b**) Feature reconstruction block for ×4 scale factor.

The FRec module is used to reconstruct the outputs of the DFE module into the final HR thermal image. There are few works that carefully design upsampling modules. We design two FRec modules for ×2/×3 and ×4 scale factors, respectively, as shown in Figure 7.

We use nearest neighbor interpolation in the FRec module to upsample the feature maps to the desired size, and leverage PAB to enhance information representation. Since the ISR task with ×4 scale factor is more burdensome, we designed the FRec module as shown in Figure 7b for the ×4 scale factor.

Overall, the generated HR thermal image output $I_{SR}$ can be expressed as:

(10) $$I_{SR}=f_{FRec}\left(x_{n}\right)+f_{up}\left(I_{LR}\right)$$

where $f_{FRec}\left(•\right)$ and $f_{up}\left(•\right)$ represent the FRec module and bilinear interpolation, respectively. We interpolate the LR thermal image to the desired size, allowing the network to learn the residual information, thereby reducing the burden and stability of the network training.

## 5. Experimental Analysis

### 5.1. Training and Testing Datasets

We use the dataset proposed by Rivadeneira et al. [47] as the training dataset. This dataset was serviced as the training and testing dataset for the PBVS [55] Thermal Image SR (TISR) challenge, which we simply abbreviate as the Challenge dataset. The Challenge dataset was created by capturing thermal images from three thermal cameras mounted on a panel. The panel was installed on the car and controlled by a developed multi-threaded script to acquire images simultaneously. The specific specifications of the three thermal cameras and the composition of the Challenge dataset are shown in Table 2 and Table 3, respectively.

Since the medium-resolution (MR) Axis and LR Domo thermal images of the Challenge dataset are not completely aligned, we adopt the Flir HR subdataset as the training dataset, and the corresponding LR counterparts are obtained by the bicubic interpolation method. As for the testing datasets, in addition to the Challenge testing dataset, in order to reflect the superior generalization of the proposed LDASRNet, we also handle Iray (http://iray.iraytek.com:7813/apply/E_Super_resolution.html/, accessed on 20 September 2023) and FLIR (https://www.flir.in/oem/adas/adas-dataset-form/, accessed on 20 September 2023) as two additional testing datasets.

### 5.2. Evaluation Metrics

PSNR and SSIM are adopted to quantitatively evaluate the performance of the proposed LDASRNet and compared models. All performance reports are evaluated on the Y channel of YCbCr color space. Following previous research [1], we crop *s* pixels around the generated $I_{SR}$, where s=2,3,4 is the corresponding scale factor.

### 5.3. Data Augmentation Method

Previous studies have shown that feature domain data augmentation (DA) methods harm the performance of ISR task, while pixel domain DA methods boost ISR accuracy. Inspired by CutBlur [56] and MPRANet [1], we adopt a mixture of DA (MoDA) strategy when training the proposed LDASRNet. Specifically, in addition to random horizontal/vertical flipping and rotation of the LR-HR pairs in each iteration during the training, one of the following pixel domain DA methods is also randomly selected to enhance the LR-HR image pairs: CutMixup [56], RGB permute [56], Blend [56], CutBlur [56], CutOut [57], CutMix [58] and Mixup [59]. Quantitative performance comparison of the proposed LDASRNet with various DA methods is shown in Table 4.

Table 4 demonstrates that the pixel domain DA methods can effectively improve ISR performance. For example, compared with the baseline model, the proposed LDASRNet trained using RGB permute or Mixup improved the PSNR metric by at least 0.09 dB, while using the CutMixup or CutBlur method improved it by 0.12 dB. Furthermore, we obtained the highest performance gains using the MoDA strategy. The results show that the pixel domain MoDA strategy in the ISR task can effectively boost accuracy.

### 5.4. Implementation Details

We train the proposed LDASRNet using the PyTorch [19] framework. We adopt the AdamW [60] optimizer instead of Adam [61] optimizer, and ablation experiments show that AdamW can slightly improve performance compared to Adam. There are 2000 epochs in total, and the initial learning rate $5\times 10^{-4}$ decreases by half every 200 epochs. The cropped ground truth resolutions corresponding to ×2, ×3 and ×4 scale factors are 96 × 96, 128 × 128 and 192 × 192, respectively.

We propose a variant network of LDASRNet named LDASRNet-T. The only difference from LDASRNet is that the non-attention branch in LDASRNet-T consists of a convolution layer with a convolution kernel size 1 × 1.

### 5.5. Ablation Experiments

**AdamW vs. Adam**. We find that LDASRNet trained with the AdamW optimizer achieves higher scores on PSNR and SSIM metrics compared to Adam. The results are shown in Table 5. We speculate that this is because AdamW directly uses the weight decay term when updating the weights.

Table 5 shows that LDASRNet trained with the AdamW optimizer on the Challenge, FLIR and Iray testing datasets achieved performance improvements of 0.01 dB, 0.04 dB and 0.08 dB, respectively, in the PSNR metric compared to Adam. Moreover, the MoDA training strategy achieves improved accuracy with AdamW or Adam optimizer, further confirming the rationality of MoDA for ISR task.

**Validity of DAB structure**. To verify the effectiveness of the proposed DAB structure, our ablation experiments compare the impact of a single path and two paths (i.e., attention branch and non-attention branch) on ISR performance. All experiments were performed on the Challenge testing dataset with the ×4 scale factor, and the results are shown in Table 6. Table 6 shows that, only equipped with non-attention branch or attention branch, the PSNR metric is 0.28 dB and 0.51 dB lower than LDASRNet, respectively. This justifies attention and non-attention branches to complement each other. Note that using only the non-attention branch results in a 0.23 dB improvement compared to using the attention branch, but the number of parameters of the former is 2.7 times that of the latter, which shows that our attention branch composed of CAB and PAB is an efficient lightweight structure.

In addition to the addition we used, the fusion methods of the two branches also include strategies such as concatenation [54] and adaptive weight [18]. We compare the performance and parameter trade-offs of the adopted addition versus concatenation and adaptive weight. As shown in case 3 and case 5 in Table 6, concatenation has 25.6 K more parameters than the additive and adaptive weight fusion methods but has the worst performance. We adopt the simplest addition strategy to achieve the best accuracy while maintaining the smallest scope of parameters.

**Model capacity**. The capacity of the model, i.e., the width and depth of the network, is critical to ISR accuracy. The number of filters in each convolutional layer in the DAB of LDASRNet is 40. In order to ablate the impact of the model capacity on performance, we propose two variant networks, LDASRNet w/Fewer Channels and LDASRNet-T. There are 32 feature channels in the DAB of LDASRNet w/Fewer Channels. The number of channels of LDASRNet-T remains the same as LDASRNet, but the kernel size of the convolutional layer in the non-attention branch is 1 × 1. Case 6 and case 8 in Table 6 show that fewer channels or a small convolution kernel in the non-attention branch is not conducive to the final thermal image SR performance. Our LDASRNet achieved optimal performance while remaining lightweight.

**Configuration of two-path structure**. We show the ablation experimental results in Table 7 to verify the impact of different configurations of the two-path structure. The two pairs of configurations, case 1 and case 5, and case 4 and case 7, show that non-attention branch equipped with CAB or PAB with dynamic attention block failed always bring positive gains. And the case 6 results indicate that dynamically assigned weights to CAB and PAB are 0.2 dB higher than the cascaded CAB and PAB structure, and the dynamic attention strategy can obtain clear performance improvements. Our non-attention branch and attention branch are supplemented with the dynamic weight structure to achieve superior accuracy.

### 5.6. Quantitative Experiments

The proposed LDASRNet is a lightweight ISR network, and we choose the following networks with parameters less than 1M for comparison: SRCNN [15], FSRCNN [16], SR-LUT [62], PAN [54], DRRN [63], $A^{2}F$ [64], AWSRN-S [65], IMDN [66], VDSR [32] and $A^{2}N$ [18]. To verify that LDASRNet has comparable or even better performance than larger networks, we select models AWSRN [65], SRMDNF [67], CARN [68], ChaSNet [44], MPRANet [1] and MDSR [26] with parameters ranging from 1.4M to 6.5M for comparison. In addition, we select RCAN [24] and EDSR [26], two networks with parameters exceeding 10M (EDSR has more than 40M parameters) as reference. The qualitative measurement results of LDASRNet and the above models on the Challenge, FLIR and Iray datasets are shown in Table 8. We show the comparison results of the three metrics PSNR, SSIM and FLOPs.

The quantitative comparison results in Table 8 show that the proposed LDASRNet achieves the highest PSNR and SSIM and the lowest complexity, i.e., the smallest FLOPs, on the three testing datasets with ×2, ×3 and ×4 scale factors. On the Challenge dataset with ×3 scale factor, for instance, LDASRNet is 0.12 dB higher than the second-best $A^{2}N$, while the scope of parameters is only about one-third of it (0.34 M vs. 1.0 M).

Surprisingly, we still achieve superior performance compared to networks with a size scope larger than LDASRNet. Specifically, the networks identified with † in Table 8 represent parameters larger than LDASRNet but the performance is worse. ChaSNet with ×4 scale factor has 14.5 M parameters, which is 41 times that of LDASRNet, and the PSNR metric is 2.16 dB lower than ours. Even compared to EDSR with more than 40M parameters, LDASRNet is only, at most, 0.2 dB lower than it on the Challenge dataset.

We also adopt an edge preservation index (EPI) metric to measure the degree of high-frequency detail recovery in the super-resolved HR thermal images. A higher EPI score indicates a higher perceived quality of the generated HR thermal image. The EPI measurement results of LDASRNet and compared models are shown in Table 9. As shown in Table 9, our LDASRNet obtains the highest EPI metric, meaning that the proposed LDASRNet can recover the most complete high-frequency details.

### 5.7. Qualitative Experiments

**Figure 8:**
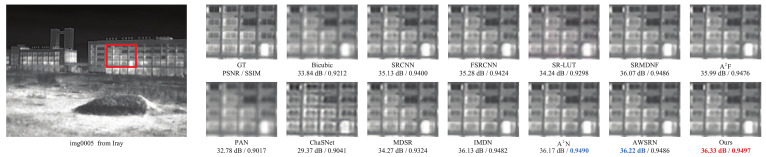
The qualitative results on the Iray testing dataset with the ×2 scale factor.

**Figure 9:**
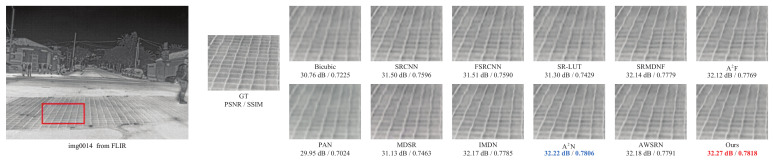
The qualitative results on the FLIR testing dataset with the ×3 scale factor.

**Figure 10:**
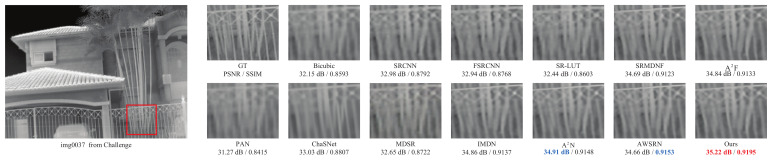
The qualitative results on the Challenge testing dataset with the ×4 scale factor.

The qualitative comparison results are shown in Figure 8, Figure 9 and Figure 10. Due to space limitations, we perform qualitative experiments on the Iray, FLIR and Challenge testing datasets with ×2, ×3 and ×4 scale factors, respectively. Note that since ChaSNet only provides model structures with ×2 and ×4 scale factors, the comparison results of ×3 exclude ChaSNet. As can be seen from Figure 8, Figure 9 and Figure 10, the proposed LDASRNet not only obtains the best PSNR and SSIM metrics, but also recovers the most complete details compared to the compared networks.

Specifically, Figure 8 shows on the thermal image *img0005* from the Iray test dataset with the ×2 scale factor that our LDASRNet achieves a PSNR metric that is 0.11 dB higher than the second-best model AWSRN, i.e., 36.33 dB vs. 36.22 dB. Similarly, Figure 9 exhibits that LDASRNet outperforms the second-best and third-best networks by 0.05 dB/0.0012 and 0.09 dB/0.0027 in terms of the PSNR/SSIM metrics, respectively, and still achieves the highest quantitative measurements. Figure 10 indicates that LDASRNet achieves the maximum performance gain on the ×4 scale factor of the Challenge test dataset for the *img0037*, with a PSNR of 35.22 dB and SSIM of 0.9195, outperforming $A^{2}N$ and AWSRN by 0.31 dB and 0.0042, respectively.

### 5.8. Compare with Lucy–Richardson–Rosen Algorithm

**Figure 11:**
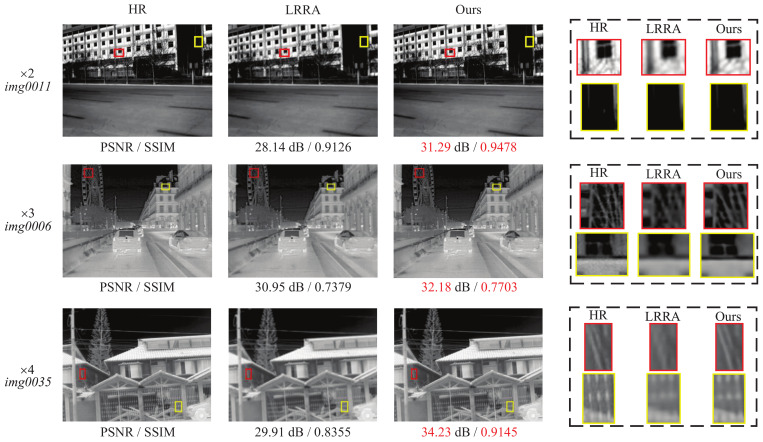
Comparison of the visual quality of thermal images processed by the proposed LDASRNet and LRRA.

In addition, we further compare the proposed LDASRNet with the recent Lucy–Richardson–Rosen algorithm (LRRA) [69,70] that exhibits superior deblurring performance. Empirically, we set the maximum number of iterations of LRRA to 8, and we use a synthetic Gaussian-type point-spread function (PSF) with standard deviation 0.3 and filter of size 3 × 3. The quantitative test results of our LDASRNet and LRRA on the ×2, ×3 and ×4 scale factors of the three test datasets of Challenge, FLIR and Iray are shown in Table 10. The comparison results of the visual perception quality of the generated images are shown in Figure 11. Note that due to space limitations, our results only show *img0011* from Iray, *img0006* from FLIR and *img0035* from Challenge.

Table 10 shows that our LDASRNet achieves the highest metrics compared to LRRA on the three test datasets with all scale factors. Specifically, on Challenge with ×2, ×3 and ×4 scale factors, our LDASRNet is 7.07 dB, 6.4 dB and 6.23 dB higher than LRRA in the PSNR metric, respectively. Similarly, on the FLIR and Iray test datasets, LDASRNet is at least 3.84 dB/0.0782 and 0.98 dB/0.4313 higher than LRRA in PSNR/SSIM metrics, respectively, i.e., 35.48 dB/0.8683 vs. 31.64 dB/0.7901 and 27.35 dB/0.8873 vs. 26.37 dB/0.8563. The results show that the proposed LDASRNet has a higher signal-to-noise ratio and more complete structural recovery than the reconstruction results of LRRA.

Qualitatively, Figure 11 shows that the HR thermal images generated by our LDASRNet possess more high-frequency details than LRRA. For example, for the buildings in *img0011* and *img0006*, the edges and textures in the results of our method are clearer, while the LRRA images are blurry. Similarly, in *img0035* in Challenge, the fence generated by LRRA obviously lacks high-frequency details and has poor perceptual quality compared to our result.

Notably, LRRA demonstrated its excellent deblurring performance in a previous study [70]. We believe that the input LR thermal image is almost free of blur, which is the main reason why LRRA is not as expected. Accordingly, comparison with deep learning-based networks and LRRA demonstrate the superior performance of the proposed LDASRNet for thermal image SR. The superior HR thermal image reconstruction accuracy and compact model size of the proposed LDASRNet show the potential for deployment in edge devices.

## 6. Conclusions

In this paper, we show that simply stacking attention modules at different depths of the deep network is suboptimal. Based on this observation, we propose a lightweight thermal image super-resolution network LDASRNet based on the dynamic attention mechanism for infrared sensors. The dynamic weight block in the proposed LDASRNet provides masks to the attention and non-attention branches according to the input features to enhance high-frequency detail extraction. Specifically, we use 1D convolution without dimensionality reduction to replace the fully connected layer to enrich the interactions between channels. The attention branch consisting of efficient channel attention and pixel attention blocks complements the non-attention branch to extract local and global features. Qualitative and quantitative experiments on three testing datasets containing various scenarios show that the proposed LDASRNet can recover high-frequency details accurately, and the lightweight structure has the potential to be deployed on edge devices.

## Figures and Tables

**Table 1:** Attention modules contribute to thermal image SR performance. **Attention module index** indicates that the *i*-th position is an attention module and the rest are residual blocks, and PSNR and SSIM are performance metrics. Red and blue text indicate best and second-best performance, respectively.

Attention Module Index	PSNR↑	SSIM↑
None	32.95	0.8078
All	33.24	0.8099
$\left\{1,2,3,4,5,6,7,8\right\}$	32.95	0.8078
$\left\{9,10,11,12,13,14,15,16\right\}$	33.24	0.8099
$\left\{2,4,6,8,10,12,14,16\right\}$	33.20	0.8089

**Table 2:** Thermal camera specifications for creating the Challenge dataset.

Image Resolution	Camera Model	FOV	Focal Length	Pixel Size	Spectral Range	Operating Temperature Range
Low (LR)	Axis Domo P1290	35.4	4 mm	12 μm	8–14 μm	−30–55 ${}^{{}^{\circ}}$C
Mid (MR)	Axis Q2901-E	35	9 mm	17 μm	8–14 μm	−40–60 ${}^{{}^{\circ}}$C
High (HR)	FC-632O FLIR	32	19 mm	17 μm	7–13.5 μm	−50–70 ${}^{{}^{\circ}}$C

**Table 3:** The structure and composition of the Challenge dataset.

Phase	Subdataset	Resolution	Amount
Training	Domo	160 × 120	951
	Axis	320 × 240	
	Flir	640 × 480	
Testing	Domo	160 × 120	50
	Axis	320 × 240	
	Flir	640 × 480	

**Table 4:** Quantitative performance comparison of the proposed LDASRNet using various data augmentation methods. We take LDASRNet trained using horizontal/vertical flipping and random rotation without the MoDA strategy as the baseline. Results are reported on the Challenge testing dataset with ×4 scale factor. Red and green text indicate the best metric and the gain relative to the baseline, respectively.

Data Augmentation Method	PSNR↑	SSIM↑
LDASRNet w/o MoDA (Baseline)	36.52 (+0.00)	0.9291 (+0.0000)
LDASRNet w/CutOut	36.62 (+0.10)	0.9300 (+0.0009)
LDASRNet w/CutMix	36.63 (+0.11)	0.9303 (+0.0012)
LDASRNet w/Mixup	36.61 (+0.09)	0.9300 (+0.0009)
LDASRNet w/CutMixup	36.64 (+0.12)	0.9309 (+0.0018)
LDASRNet w/RGB permute	36.61 (+0.09)	0.9299 (+0.0008)
LDASRNet w/Blend	36.60 (+0.08)	0.9298 (+0.0007)
LDASRNet w/CutBlur	36.64 (+0.12)	0.9310 (+0.0019)
**LDASRNet w/MoDA (Our)**	36.65 (+0.13)	0.9312 (+0.0021)

**Table 5:** Performance comparison between AdamW and Adam optimizers. All results are reported on the ×2 scale factor. Red text indicates the best metrics.

Adam	AdamW	MoDA	Challenge Dataset		FLIR Dataset		Iray Dataset	
			PSNR↑	SSIM↑	PSNR↑	SSIM↑	PSNR↑	SSIM↑
√			44.18	0.9774	35.29	0.8583	33.26	0.9358
	√		44.20	0.9788	35.33			0.9376
√		√	44.33	0.9785	35.44	0.8689	33.40	0.9384
	√	√	44.34	0.9870	35.48	0.8683	33.48	0.9466

**Table 6:** Ablation studies: effects of different DAB structural configurations. **LDASRNet w/Fewer Channels** means that the number of channels of the feature map in DAB is reduced to 32. All experiments were performed on the Challenge testing dataset with the ×4 scale factor. Red text indicates the best metrics.

		Path		Path Fusion					Params (*K*)	Metric
		Non-Attention	Attention	Addition	Concatenation	Adaptive-Weight	LDANet (1 × 1 conv)	LDANet (3 × 3 conv)		PSNR↑
Single Path	LDASRNet w/Non-Attention	√							323.299	36.37
	LDASRNet w/Attention		√						119.187	36.14
Two Paths w/o DAB	LDASRNet w/Addition	√	√	√					349.587	36.50
	LDASRNet w/Concatenation	√	√		√				375.187	36.47
	LDASRNet w/Adaptive-Weight	√	√			√			349.587	36.46
LDASRNet	LDASRNet w/Fewer Channels	√	√					√	232.811	36.58
	LDASRNet-T	√	√				√		146.131	36.60
	LDASRNet	√	√					√	350.931	36.65

**Table 7:** Ablation studies: effects of various structural configurations of two paths. **C-P AB** means channel- and pixel-wise attention block. All experiments were performed on the Challenge testing dataset with ×4 scale factor. Red text indicates the best metric.

Settings					Params (K)	Metric	Gains from DAB
Non-Attention	CAB	PAB	C-P AB	DAB		PSNR↑	PSNR↑
√	√				323.347	36.40	–
			√		119.187	36.17	–
√			√		349.587	36.53	–
√		√			349.539	36.51	–
√	√			√	324.691	36.38	+0.02
			√	√	120.531	36.19	−0.02
√		√		√	350.883	36.60	+0.09
√			√	√	350.931	36.65	+0.12

**Table 8:** Quantitative comparison results of **PSNR/SSIM/FLOPs** metrics. Red and blue texts indicate the best and second-best performance, respectively (except those with parameters greater than 10 M). † indicates that the model parameters are larger than our LDASRNet but the accuracy is worse.

Scale	Size Scope	Network	Prams	Testing Datasets		
				Challenge	FLIR	Iray
×2	≤1 M	FSRCNN	0.012 M	42.11/0.9841/0.0010T	34.95/0.8637/0.0010T	32.20/0.9361/0.0003T
		SR-LUT	0.017 M	41.23/0.9783/0.0039T	34.49/0.8563/0.0041T	30.96/0.9200/0.0014T
		SRCNN	0.057 M	39.22/0.9790/0.0176T	34.79/0.8607/0.0187T	31.83/0.9314/0.0063T
		PAN	0.1 M	39.06/0.9649/0.0142T	33.25/0.8205/0.0152T	28.85/0.8804/0.0051T
		DRRN	0.3 M	43.44/0.9848/0.8161T	35.34/0.8663/0.8705T	32.92/0.9423/0.2938T
		$A^{2}F$	0.3 M	44.26/0.9868/0.0236T	35.43/0.8676/0.0251T	33.25/0.9451/0.0085T
		AWSRN-S	0.4 M	43.89/0.9861/0.0304T	35.38/0.8670/0.0324T	33.03/0.9438/0.0109T
		IMDN	0.7 M	44.12/0.9861/0.0015T	35.46/0.8672/0.0016T	33.36/0.9451/0.0006T
		VDSR	0.7 M	43.77/0.9868/0.2042T	35.36/0.8679/0.2178T	32.89/0.9438/0.0735T
		$A^{2}N$	1.0 M	44.23/0.9867/0.0826T	35.47/0.8679/0.0881T	33.28/0.9450/0.0297T
		Ours	0.34 M	44.34/0.9870/0.0295T	35.48/0.8683/0.0315T	33.48/0.9466/0.0106T
	<7 M	AWSRN	1.4 M	44.38/0.9871/0.1068T	35.53/0.8680/0.1140T	33.60/0.9474/0.0385T
		SRMDNF †	1.5 M	44.14/0.9869/0.1146T	35.41/0.8671/0.1222T	33.18/0.9445/0.0412T
		CARN †	1.6 M	43.97/0.9867/0.0743T	35.43/0.8676/0.0792T	33.35/0.9459/0.0267T
		ChaSNet †	3.2 M	32.73/0.9564/0.2433T	30.27/0.8184/0.2592T	25.93/0.8580/0.0876T
		MPRANet	4.4 M	45.50/0.9903/0.3892T	35.88/0.8857/0.4152T	33.99/0.9537/0.1401T
		MDSR †	6.5 M	40.66/0.9804/0.4993T	34.40/0.8573/0.5325T	30.49/0.9168/0.0799T
	>10 M	RCAN	15.4 M	44.50/0.9873/1.1766T	35.63/0.8697/1.2708T	33.91/0.9496/0.4289T
		EDSR	40.7 M	44.44/0.9872/3.1282T	35.56/0.8687/3.3368T	33.81/0.9490/1.1261T
×3	≤1 M	FSRCNN	0.012 M	37.68/0.9487/0.0004T	32.15/0.7958/0.0003T	28.29/0.8562/0.0002T
		SR-LUT	0.017 M	37.12/0.9410/0.0017T	32.14/0.7858/0.0019T	27.79/0.8415/0.0006T
		SRCNN	0.057 M	37.68/0.9505/0.0176T	32.54/0.7980/0.0186T	28.32/0.8584/0.0063T
		PAN	0.1 M	35.00/0.9249/0.0089T	31.08/0.7582/0.0094T	26.43/0.8038/0.0032T
		DRRN	0.3 M	38.78/0.9586/0.8148T	33.07/0.8075/0.8657T	29.22/0.8811/0.2938T
		$A^{2}F$	0.3 M	39.38/0.9617/0.0105T	33.15/0.8082/0.0112T	29.39/0.8848/0.0038T
		AWSRN-S	0.5 M	38.97/0.9549/0.0162T	33.05/0.8067/0.0172T	29.19/0.8819/0.0058T
		IMDN	0.7 M	39.38/0.9621/0.0007T	33.17/0.8082/0.0008T	29.34/0.8858/0.0003T
		VDSR	0.7 M	38.76/0.9594/0.2039T	35.03/0.8075/0.2166T	29.17/0.8814/0.0735T
		$A^{2}N$	1.0 M	39.44/0.9620/0.0392T	33.17/0.8086/0.0417T	29.41/0.8854/0.0141T
		Ours	0.34 M	39.56/0.9628/0.0157T	33.24/0.8099/0.0167T	29.50/0.8873/0.0057T
	<7 M	AWSRN	1.5 M	39.60/0.9630/0.05101T	33.24/0.8103/0.0532T	29.64/0.8912/0.0181T
		SRMDNF †	1.5 M	39.14/0.9603/0.0514T	33.10/0.8075/0.0546T	29.33/0.8841/0.0185T
		CARN †	1.6 M	39.31/0.9617/0.0395T	33.18/0.8092/0.0420T	29.49/0.8878/0.0143T
		ChaSNet	–	–	–	–
		MPRANet	4.4 M	40.72/0.9741/0.2113T	33.34/0.8190/0.2245T	30.62/0.9092/0.0762T
		MDSR	6.7 M	36.67/0.9942/0.1365T	32.00/0.7881/0.1456T	27.40/0.8361/0.0463T
	>10 M	RCAN	15.6 M	39.87/0.9646/0.5287T	33.33/0.8120/0.5617T	30.00/0.8966/0.1906T
		EDSR	43.7 M	39.76/0.9640/1.4899T	33.30/0.8118/1.5830T	24.73/0.6140/0.5372T
×4	≤1 M	FSRCNN	0.012 M	35.11/0.9102/0.0002T	31.01/0.7439/0.0003T	26.58/0.7922/0.0001T
		SR-LUT	0.017 M	34.59/0.8994/0.0010T	30.64/0.7308/0.0011T	26.10/0.7748/0.0003T
		SRCNN	0.057 M	35.05/0.9107/0.0175T	30.96/0.7435/0.0187T	26.45/0.7901/0.0063T
		PAN	0.2 M	33.18/0.8813/0.0071T	29.79/0.7029/0.0075T	24.97/0.7353/0.0025T
		DRRN	0.3 M	35.97/0.9139/0.8161T	31.48/0.7574/0.8705T	27.14/0.8176/0.2938T
		$A^{2}F$	0.3 M	36.45/0.9289/0.0060T	31.59/0.7603/0.0064T	27.36/0.8266/0.0022T
		AWSRN-S	0.6 M	36.21/0.9263/0.0112T	31.51/0.7585/0.0120T	27.21/0.8210/0.0040T
		IMDN	0.7 M	36.51/0.9290/0.0005T	31.62/0.7607/0.0005T	27.31/0.8275/0.0002T
		VDSR	0.7 M	35.98/0.9249/0.2042T	31.46/0.7579/0.2178T	27.15/0.8197/0.0735T
		$A^{2}N$	1.0 M	36.40/0.9280/0.0242T	31.58/0.7592/0.0258T	27.23/0.8233/0.0087T
		Ours	0.35 M	36.65/0.9312/0.0109T	31.68/0.7625/0.0116T	27.35/0.8296/0.0039T
	<7 M	AWSRN †	1.6 M	36.37/0.9316/0.0304T	31.67/0.7630/0.0324T	27.53/0.8343/0.0109T
		SRMDNF †	1.5 M	36.25/0.9270/0.0294T	31.53/0.7591/0.0314T	27.33/0.8260/0.0106T
		CARN †	1.6 M	36.48/0.9296/0.0303T	31.63/0.7616/0.0323T	27.40/0.8292/0.0109T
		ChaSNet †	14.5 M	34.49/0.8957/0.2769T	30.55/0.7162/0.2954T	26.25/0.7722/0.0997T
		MPRANet	4.41 M	36.95/0.9365/0.1494T	31.92/0.7700/0.1593T	27.82/0.8488/0.0538T
		MDSR †	6.7 M	34.23/0.9032/0.1366T	30.51/0.7336/0.1456T	27.40/0.8361/0.0492T
	>10 M	RCAN	15.6 M	36.94/0.9244/0.2978T	31.74/0.7647/0.3177T	27.72/0.8407/0.1072T
		EDSR	43.7 M	36.81/0.9331/1.4899T	33.30/0.8118/1.5830T	27.70/0.7922/0.5372T

**Table 9:** Edge preservation index (EPI) metric measurement. All experiments were performed on the Challenge testing dataset with ×2 scale factor. Red and blue text represent the best and second-best metrics respectively.

Model	Bicubic	FSRCNN	SR-LUT	SRCNN	PAN	$A^{2}F$	IMDN	$A^{2}N$	AWSRN	SRMDNF	ChaSNet	MDSR	Ours
EPI↑	0.7549	0.8069	0.6820	0.7743	0.7587	0.8456	0.8442	0.8470	0.8478	0.8428	0.7502	0.7517	0.8497

**Table 10:** Quantitative comparison results of the proposed LDASRNet and LRRA. PSNR (dB) and SSIM are the metrics.

Scale	Method	Testing Datasets		
		Challenge	FLIR	Iray
×2	LRRA	37.27/0.9673	31.64/0.7901	32.35/0.9119
	Ours	44.34/0.9870	35.48/0.8683	33.48/0.9466
×3	LRRA	33.16/0.9086	29.36/0.6867	28.30/0.8563
	Ours	39.56/0.9628	33.24/0.8099	29.50/0.8873
×4	LRRA	30.42/0.8452	27.80/0.6060	26.37/0.7883
	Ours	36.65/0.9312	31.68/0.7625	27.35/0.8296

## Data Availability

The data that support the findings of this study are available on request from the corresponding author.
